# Completely Anonymous Multi-Recipient Signcryption Scheme with Public Verification

**DOI:** 10.1371/journal.pone.0063562

**Published:** 2013-05-10

**Authors:** Liaojun Pang, Huixian Li, Lu Gao, Yumin Wang

**Affiliations:** 1 School of Life Sciences and Technology, Xidian University, Xi’an, China; 2 Department of Computer Science, Wayne State University, Detroit, Michigan, United States of America; 3 School of Computer Science and Engineering, Northwestern Polytechnical University, Xi’an, China; University of Catania, Italy

## Abstract

Most of the existing multi-recipient signcryption schemes do not take the anonymity of recipients into consideration because the list of the identities of all recipients must be included in the ciphertext as a necessary element for decryption. Although the signer’s anonymity has been taken into account in several alternative schemes, these schemes often suffer from the *cross-comparison attack* and *joint conspiracy attack*. That is to say, there are few schemes that can achieve complete anonymity for both the signer and the recipient. However, in many practical applications, such as network conference, both the signer’s and the recipient’s anonymity should be considered carefully. Motivated by these concerns, we propose a novel multi-recipient signcryption scheme with complete anonymity. The new scheme can achieve both the signer’s and the recipient’s anonymity at the same time. Each recipient can easily judge whether the received ciphertext is from an authorized source, but cannot determine the real identity of the sender, and at the same time, each participant can easily check decryption permission, but cannot determine the identity of any other recipient. The scheme also provides a public verification method which enables anyone to publicly verify the validity of the ciphertext. Analyses show that the proposed scheme is more efficient in terms of computation complexity and ciphertext length and possesses more advantages than existing schemes, which makes it suitable for practical applications. The proposed scheme could be used for network conferences, paid-TV or DVD broadcasting applications to solve the secure communication problem without violating the privacy of each participant.

Key words: Multi-recipient signcryption; Signcryption; Complete Anonymity; Public verification.

## Introduction

With development of network technology and its applications, a lot of group-oriented network services such as network multicasting or broadcasting have been proposed. Usually, in these services, a message sender is required to securely send the same messages to a group of recipients, such that only a certain number of recipients can read the messages while unauthorized recipients can extract nothing useful from these messages [1]. Therefore, the concept of multi-recipient encryption was put forward [2]–[6], and it has been considered as one of most promising solutions to solve the security problem of securing multicasting or broadcasting. Later, combining the concept of multi-recipient encryption with the idea of Zheng’s signcryption [7], Duan *et al.* [8] proposed the first multi-recipient signcryption scheme. In their scheme, to achieve the goal of sending the same message to all authorized recipients confidentially, the sender only needs to execute one signcryption operation, and at the same time, each recipient can verify the validity of messages. Since then, many excellent multi-recipient signcryption schemes [9]–[11] were proposed, which take more security properties into consideration than Duan *et al*.’s scheme. In general, multi-recipient signcryption can be used in many important applications, such as paid-TV or DVD broadcasting systems [10], where only authorized or paying users should be able to access such services.

Nevertheless, today, more and more people are concerned regarding personal privacy, thus participant anonymity should be taken into account when designing multi-recipient signcryption [12]. For example, in paid-TV and DVD broadcasting application systems, service providers do not want others to obtain the real identities from the ciphertext messages. Therefore, multi-recipient signcryption with the sender (or called the signer) anonymity had been introduced. In literature, there have been several multi-recipient signcryption schemes [13]–[17] which try to assure anonymity of the sender. The concept of anonymous signature was firstly proposed by Rivest *et al*. [18]. In 2005, Huang *et al*. [19] proposed the first anonymous signcryption scheme, which used an ID-based ring signature to assure anonymity of the signer. However, their scheme is only a single-recipient scheme. Later, based on similar thoughts, Lal *et al*. [13] extended this method for multi-recipient environments. Furthermore, a multi-recipient scheme with anonymity of the sender [14]–[17] was proposed. Although these schemes [13]–[17] provide solutions for assuring signer anonymity, there are still some unsolved issues. For example, they suffer from two new attacks known as the *cross-comparison attack* [20] and the *joint conspiracy attack* [21]. Based on the ring signature, schemes [13]–[17] construct a list which includes the real signer and several valid participants which are chosen randomly by the signer hiding the real signer in this list. Although this perfectly works to some extent, an attacker can obtain a number of different ciphertexts from the same message source by closely monitoring network traffic, thus by comparing the signers’ identities from different lists an attacker can narrow down the scope of the target signer. Using this scheme, an attacker can directly obtain the identity of the real signer. Even if the attacker does not directly obtain the real signer’s identity, he/she has narrowed down the scope of the attacker’s guess. In addition, it is still possible for such an attacker to retrieve a list which includes the real signer. Then, he/she can cooperate with some participants in the list to narrow down the scope and guess the real signer with a larger probability. In addition, the list of chosen participants can increase the length of the ciphertext quite significantly, potentially reducing the transmission efficiency. More important, the identities of all the authorized recipients are usually included in the ciphertext of these anonymous schemes in plaintext [13]–[19], which is not always wanted.

Generally speaking, anonymity of participants includes both the sender’s and the recipient’s anonymity. Besides the anonymity of the sender, the anonymity of the recipient is often equally important so that designers of multi-recipient signcryption schemes should pay attention. For example, in paid-TV and DVD broadcasting application systems, no user should accept that his/her subscription of these services is publicly viewable to others especially when the service is quite sensitive. However, unfortunately, almost none of the existing schemes take the anonymity of recipients into consideration because the identity of each recipient must be included in the ciphertext as a necessary element for decryption. The list of the authorized recipients’ identities in the ciphertext is used to show who are the authorized recipients and how each authorized recipient gets his/her person-specific data for encryption from the ciphertext during the decryption process. Thus, schemes [9]–[11] directly expose the recipient’s identity and therefore violate their privacy. Also, the fact that different recipients have different person-specific data for decryption can lead to decryption unfairness. This means that if some recipient’s person-specific data are damaged due to communication errors, he/she cannot decrypt the ciphertext but the others can still decrypt the ciphertext correctly [12]. Therefore, it is urgent and challenging for researchers to solve the recipient anonymity issue of multi-recipient signcryption.

Following the arguments above, it is known that almost none of the existing multi-recipient signcryption schemes take the full anonymity of recipients and senders into account. Although there are several schemes that provide a solution for anonymity of the signer, they are not perfect, that is, they suffer from the *cross-comparison attack* and the *joint conspiracy attack*. Therefore, existing schemes cannot deal with the anonymity of the sender or the recipient properly. Furthermore, these schemes are not suitable for applications that need complete anonymity for the sender and the recipient. For example, in a network conference application, every conference participant often wants to be kept anonymous when he/she is taking part in the conference discussion. Furthermore, if a participant (i.e. sender) wants to publish criticism or objections, he/she hopes that others (i.e. recipients) do not know his/her identity. At the same time, the recipient cannot want the other recipients to reveal that he/she is an authorized recipient. In fact, today, anonymity is one of the most important prerequisites for people to talk freely and make objective decisions.

Motivated by the above, this paper proposes a completely anonymous multi-recipient signcryption scheme which meets: (1) The identity of the sender is kept secret; (2) The identities of all the recipients are kept secret; (3) Each recipient can easily judge whether the received message is from an authorized source, but he/she cannot determine the real identity of the sender; (4) Each recipient can easily judge whether he/she is an authorized recipient, but he/she cannot determine the identity of any other authorized recipient; (5) The validity of ciphertext can be verified publicly. Speaking of practical applications, the proposed scheme can be in principle used for network conference, paid-TV or DVD broadcasting application systems to assure secure communication among authorized participants, while at the same time, providing complete anonymity for all participants.

To facilitate the description of our scheme, notations used throughout the document are summarized in Table 1.

**Table 1:** Notations.

Name	meaning
*q*	Large prime integer
*G*_1_	Additive group of order *q*
*G*_2_	Multiplicative group of order *q*
*Z_q_* ^*^	The set of positive integers which are less than *q*
*P*	Generator of *G*_1_
*e*	Bilinear mapping, i.e. *e*:*G*_1_×*G*_1_→*G*_2_
*H_i_*	Cryptographic hash function, *i* = 1, 2, 3, 4
*ID_i_*	The identity of the participant *i*
*Q_i_*	Public key of *ID_i_*
*D_i_*	Private key of *ID_i_*
PKG	Private key generator
*s*	The master key of PKG
*P_pub_*	The system master public key
CDH	Computational Diffie-Hellman Problem
DBDH	Decisional Bilinear Diffie-Hellman Problem
DBDH-M	Modified Decisional Bilinear Diffie-Hellman Problem
PPT	Probabilistic polynomial time
*Pr*	The probability of an event
*Adv*	The advantage of one algorithm in solving a problem

## Preliminaries

### Complexity Assumptions

The security of the proposed scheme is based on the following problems and security assumptions.

Let *G*_1_ and *G*_2_ be two cyclic groups of prime order *q* and let *P* be a generator of *G*_1_. Let *e*: *G*_1_×*G*_1_→*G*_2_ be a bilinear mapping. The DBDH, CDH and DBDH-M problems can thus be defined as:

1. **Decisional Bilinear Diffie-Hellman (DBDH) Problem:** Given 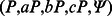, for 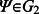 and unknown 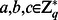, determine whether 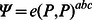 holds.
**Definition 1:** *The advantage of any probabilistic polynomial time (PPT) algorithm* B *in solving the DBDH Problem is defined as: 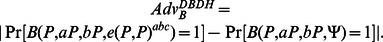
*
**DBDH assumption**: For any PPT algorithm *B*, 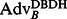 is negligible.
2. **Computational Diffie-Hellman (CDH) Problem**: Given 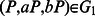, for some 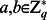, compute *abP*.
**Definition** ***2:*** *The advantage of any PPT algorithm* B *in solving the CDH Problem is defined as: 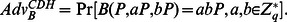
*
**CDH assumption**: For any PPT algorithm *B*, 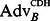 is negligible.
3. **Modified Decisional Bilinear Diffie-Hellman (DBDH-M) Problem:** Given 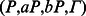, for 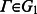 and unknown 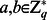, determine whether 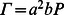 holds.
**Definition 3:** *The advantage of any PPT algorithm* B *in solving the* DBDH-M Problem *is defined as: 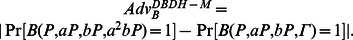
*
**DBDH-M assumption**: For any PPT algorithm *B*, 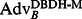 is negligible.

### Algorithm Model

Our identity(ID)-based multi-recipient signcryption scheme with complete anonymity consists of four algorithms, namely: Setup, Extract, Anony-signcrypt and De-signcrypt, shown as follows:

#### Setup

Private Key Generator (PKG) runs this algorithm to generate a master key *s* and public parameters *params*. Note that the public parameters are publicly known while the master key must be kept secret.

#### Extract

This algorithm is run by PKG to extract the private key of the user. With a user’s identity ID, PKG’s master key *s* and the public parameter *params* as input, it outputs the private key *D* associated with ID, namely *D* = Extract(ID, *s*, *params*). The private key *D* must be kept secret.

#### Anony-signcrypt

This algorithm is run by the signer ID*_S_*. With PKG’s public parameter *params*, a plaintext message *M*, a list of recipients’ identity *L* = {ID_1_,ID_2_,…,ID*_n_*} as input, the signer ID*_S_* runs this algorithm to generate a ciphertext *C* associated with *M*, namely *C* = Anony-signcrypt (*params*, *M*, *L*, *D_S_*), which satisfies *L
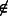
C* and ID*_S
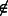
_C*.

#### De-signcrypt

With the ciphertext *C*, PKG’s public parameter *params*, the recipient’s identity ID*_i_*(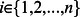) and its private key *D_i_* as input, the recipient can run this algorithm to decrypt the ciphertext. The recipient can first judge whether he/she is an authorized recipient. If not, he/she outputs an error message 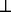 and exits the algorithm. Otherwise, he/she continues to carry out the decryption process and outputs the plaintext *M* associated with *C*, namely *M* = De-signcrypt (*C*, *params*, *D_i_*).

### Message Confidentiality

The security model of ciphertext indistinguishability under chosen ciphertext attack was first proposed by Canetti *et al*. [20]. Later, Duan *et al*. [8] extended this security model for the multi-recipient environments, called as indistinguishability of ciphertexts under selective multi-ID, chosen ciphertext attack (IND-sMIBSC-CCA) shown as Definition 4.

#### Definition 4

IND-sMIBSC-CCA: Let A be a polynomial-time attacker and 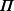 be an ID-based multi-recipient scheme. Consider that A interacts with a Challenger B in the following game:

#### Setup

Challenger B runs this algorithm to generate master key *s* and public parameters params, sends params to A, and keeps the master key *s* secret. Upon receiving public parameters, A outputs n target identities 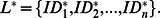


#### Phase 1

A performs a number of queries to B:

Extraction query: Upon receiving private key extraction query about an identity ID, 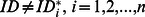, B runs the Extract algorithm to get D = Extract(ID, *s*, params).

Anony-signcrypt*ion* query: A chooses a target plaintext M, a list of recipients’ identity information *L = {ID_1_,ID_2_,…,ID_n_}* and gives them to B. B randomly chooses an identity *ID_S_*, computes the private key *D_S_*, and generates the ciphertext C = Anony-signcrypt (params,M,L, *D_S_*) and returns it to A.

De-signcrypt*ion* query: *A generates the list of target identities* 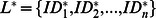 and a ciphertext C. B randomly chooses an identity 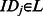 and computes its private key D_j_. *I*f C is a valid ciphertext, B decrypts it to obtain the corresponding plaintext M = De-signcrypt (C, params, D_j_) and returns it to A; otherwise, B outputs an error message 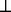.

#### Challenge

A outputs a target plaintext pair (M_0_, M_1_) and an arbitrary identity *ID_S_* with its private key *D_S_*. Upon receiving (M_0_, M_1_) and *D_S_*, B picks *up a random bit* 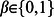 and creates a target ciphertext 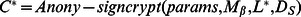, and then returns C^*^ to A.

#### Phase 2

A performs a number of queries like Phase 1. Note that A cannot query the identity information in L^*^ in the Extraction query, and cannot query C^*^ in the De-signcryption query.

#### Guess

Finally, A outputs its guess 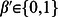. If 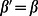, he wins this game.

An attacker *A* mentioned above is referred to as an IND-sMIBSC-CCA attacker. We define *A*’s guessing advantage as follows:
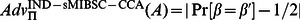
(1)


The scheme 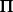 is said to be 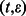-IND-sMIBSC-CCA secure, if for any IND-sMIBSC-CCA attacker *A*, its guessing advantage is less than 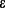 within polynomial running time *t*.

### Unforgeability

This security model has been proposed by Duan *et al*. [8] and is called strong existential unforgeability under selective multi-ID, chosen message attack (SUF-MIBSC-CMA) shown as Definition 5.

#### Definition 5

SUF-sMIBSC-CMA: Suppose F is a forger, and let 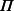 be an ID-based multi-recipient scheme. Consider that F interacts with a Challenger B in the following game:

#### Setup

B runs this algorithm to generate a master key *s* and a public parameter params. B gives the params to F and keeps *s* secretly. Upon receiving this parameter, F outputs n target identities 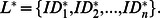


#### Attack

F performs a number of queries to B as described in Definition 4.

#### Forgery

F finally outputs a new ciphertext message C^*^, a list of recipient identities *L = {ID_1_,ID_2_,…,ID_n_}*. If C^*^ is the ciphertext of the message M generated by 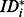 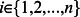 and can be decrypted by any of recipients in *L*, C^*^ is a valid ciphertext and F wins this game. The restriction here is that F cannot ask for private key extraction on 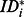, and C^*^ cannot be produced by the Anony-signcrypt algorithm.

The scheme 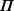 is said to be 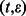-SUF-sMIBSC-CMA secure, if for any SUF-sMIBSC-CMA attacker *F*, its guessing advantage is less than 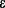 within polynomial running time *t*.

### Recipient Anonymity

This security model has been proposed by Fan *et al*. [5] and is called anonymous indistinguishability of encryptions under selective ID, chosen ciphertext attack (ANON-sID-CCA) and shown as Definition 6.

#### Definition 6

ANON-sID-CCA: Let A be a polynomial-time attacker. Let 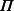 be an ID-based multi-recipient scheme. Consider that A interacts with a Challenger B in the following game:

#### Setup

Challenger B runs the Setup algorithm to generate the master key s and public parameters params. Then, B sends params to A and keeps s secret.

#### Phase 1

A outputs a target identity pair (ID_1_^*^, ID_2_^*^). Upon receiving (ID_1_^*^, ID_2_^*^), the Challenger B randomly chooses 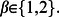


#### Phase 2

A issues private key extraction queries. Upon receiving a private key extraction query, denoted by ID_j_, Challenger B runs the private key extraction algorithm to get D_j_ = Extract(ID_j_, s, params). The constraint here is that 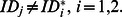


#### Phase 3

A issues de-signcryption queries for the target identities. Upon receiving a de-signcryption query about (C^*^, ID_i_^*^), i = 1,2, Challenger B returns M^*^ = De-signcrypt (C^*^,params,D_i_^*^) to A, where D_i_^*^is the private key of ID_i_^*^.

#### Challenge

A outputs a target plaintext M to B. Then, Challenger B creates a related ciphertext 
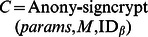
 and returns it to A.

#### Phase 4

A issues private key extraction queries as those in Phase 2 and de-signcryption queries for target identities as those in Phase 3. The restriction here is that 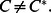


#### Guess

Finally, A outputs its guess 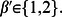 If 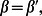 A wins the game.

An attacker *A* mentioned above is referred to as an ANON-sID-CCA attacker. We define *A*’s guessing advantage as follows:
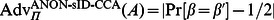
(2)


The scheme 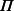 is said to be 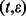-ANON-sID-CCA secure, if for any ANON-sID-CCA attacker *A*, its guessing advantage is less than 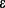 within polynomial running time *t*.

## Methods

The proposed scheme is composed of the following four algorithms. And at the same time, we shall take the network conference application as an example to show how to use our scheme.

### Setup Algorithm

PKG performs the following process:

1. Let *G*_1_ be an additive group and *G*_2_ be a multiplicative group with the same prime order *q*, (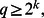 *k* is a long integer). Let *P* be a generator of *G*_1_. Choose a bilinear mapping *e*: *G*_1_×*G*_1_→*G*_2_.
2. Define four one-way hash functions: *H*_1_:{0,1}^*^→*G*_1_; *H*_2_: *G*_2_→{0,1}^|*M*|^; *H*_3_:{0,1}^*^→*Z_q_* ^*^; *H*_4_:{0,1}^|*M*|^×*G*_1_×…×*G*_1_→*Z_q_* ^*^, where |*M*| is the length of the plaintext message.
3. Choose a random number 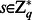 as the master key, and set 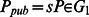 as the system’s public key. Publish the system parameter *params = *<*G*_1_,*G*_2_,*q*,*e*,*P*,*P_pub_*,*H*_1_,*H*_2_,*H*_3_,*H*_4_> and keep the master key *s* secret.

Practically speaking, PKG is acted by some authority. For example, in a network conference application, the organizer of a conference should deal with the PKG, which is responsible for developing the system parameters as the steps mentioned above.

### Extract Algorithm

With *params*, *s*, and an identity 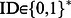 as input, PKG performs this algorithm to generate the private key of the identity ID:

1. Compute ID’s public key *Q*_ID_ = *H*_1_(ID).
2. Compute ID’s private key *D*_ID_ = *sH*_1_(ID) = *sQ*_ID_.

Each participant, the sender or the recipient, should register himself/herself at PKG and obtain his/her private key from PKG by this algorithm. For example, in a network conference application, if someone wants to attend a conference and talk with other participants, he/she must firstly send his/her ID information to the organizer PKG to get his/her own private key computed by PKG.

### Anony-signcrypt Algorithm

With *params*, a plaintext *M* and his/her private key *D_S_* as input, the signer ID*_S_* chooses a list of recipients’ identity *L* = {ID_1_,ID_2_,…,ID*_n_*} and performs this algorithm to generate the ciphertext *C* of the plaintext *M*:

1. Randomly choose two secret integers 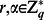 and a secret element 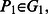 and then compute 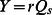, 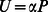, 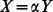, 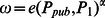 and 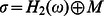, where *Q_S_* is the public key of ID*_S_*.
2. For *i* = 1,2,…,*n*, compute *x_i_ = H_3_*(ID_*i*_) and 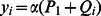, where Q_i_ is the public key of ID_*i*_.
3. For *i* = 1,2, …,*n*, compute 
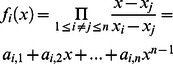
, where 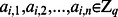.
4. For *i* = 1, 2, …, *n*, compute 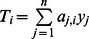 and then let *T* = {*T_1_,T_2_,…,T_n_*}.
5. Compute 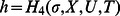, and then compute 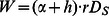, where D_*S*_ is the private key of ID_S_.
6. Generate the ciphertext: 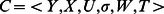.

After obtaining the private key, each participant can securely and anonymously send messages to other participants that he/she selects. For example, in a network conference application, any participant can freely select some participants as expected recipients to receive his/her messages. What he/she needs to do is to encrypt the messages by this algorithm and then broadcast the ciphertext.

### De-signcrypt Algorithm

The algorithm is carried out by the recipient. With 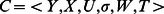, *params*, the recipient’s identity ID*_i_* and his/her private key *D_i_* as input, the recipient ID*_i_* decrypts *C* as follows:

#### Public verification

The one, who has not registered himself/herself with PKG to get his/her private key, can use the following steps to check the integrity or validity of the ciphertext. The registered participant can skip this process and directly jump to the following judgement algorithm:

1. Compute 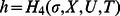.
2. Verify whether the equation *e*(*W*, *P*) = *e*(*X*+*hY*, *P_pub_*) holds. If yes, the ciphertext is valid. Otherwise, the ciphertext is invalid or has been damaged during transmission.

#### Judgement

The one, who has registered himself/herself with PKG to get his/her private key, can use the following steps to check whether the ciphertext is valid and whether he/she is an authorized recipient before the following encryption process:

1. Compute 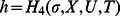.
2. Check whether the equation *e*(*W*, *Q_i_*) = *e*(*X*+*hY*, *D_i_*) holds. If yes, it means that ID*_i_* is one of the recipients designated by the signer and the ciphertext is valid. Otherwise, the recipient quits the decryption process.

#### De-signcryption

The authorized user can recover the plaintext by the following steps:

1. Compute *x_i_* = *H*_4_(ID*_i_*) and then compute 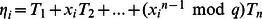.
2. Compute 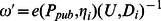 and then get the plaintext as 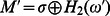.

The one who receives the broadcasting ciphertext can verify the validity of the message and judge whether he/she is authorized by the public verification or judgement algorithm. If necessary, he/she can use the de-signcrypt algorithm to decrypt the ciphertext. In a network conference application, due to the nature of the broadcast communication, anyone, authorized recipients or unauthorized ones, can easily receive the ciphertext and check the validity of the message and the authorization of the decryption. But, only the authorized recipients can decrypt it correctly.

## Results and Discussion

### Correctness Analyses

#### Theorem 1

The public verification algorithm in the De-signcrypt algorithm is correct.

#### Proof



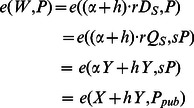
(3)In our scheme, although the identity of the real signer is not included in the ciphertext, his/her private key is definitely necessary in the signcryption process, which ensures that only legal participants who have registered himself/herself with PKG can generate a valid ciphertext. That is to say, through this algorithm, anyone can check whether a ciphertext is generated by an authorized participant, but he/she cannot determine the real identity of the signer.

#### Theorem 2

*The judgement algorithm* in the De-signcrypt algorithm *is correct.*

#### Proof

For each authorized ID*_i_* where 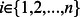, we have



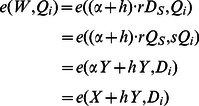
(4)Similarly, because the private key of the real signer is necessary in the signcryption process, this algorithm can also be used to check the validity of ciphertext. At the same time, this algorithm can help a participant, who has registered himself/herself with PKG, to judge whether himself/herself is an authorized recipient, because the private key of the recipient is also necessary in the judgement.

#### Theorem 3

*The decryption algorithm* in the De-signcrypt algorithm *is correct.*

#### Proof

The authorized ID*_i_*, 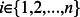, can compute 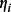 as follows:
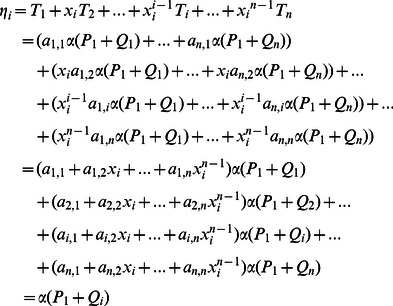
(5)


Thus, we can get 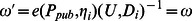, because
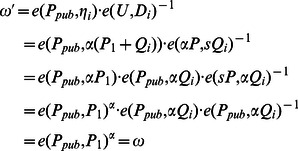
(6)


Then, we can get the plaintext through the computations 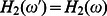 and 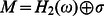.

### Security Analyses

We shall give security proof of the proposed scheme on confidentiality, unforgeability and anonymity under the random oracle model.

#### Theorem 4

*In the* IND-sMIBSC-CCA *security model, if an adversary A has an advantage* 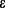 *against the game defined in Definition 4 within running time t (where* A mak*es* at most q_e_ private key extraction queries, q_s_ *anony-signcryption queries, q_d_* de*-sign*cryption queries and 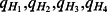 queries to the Hash functions H_1_, H_2_, H_3_ and H_4_, *respectively), then there is a algorithm* B *in solving the DBDH problem in the time* 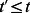 *with an advantage* 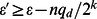.

#### Proof

The challenger *B* is challenged with an instance 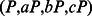 of the DBDH problem. Assume that there is an adversary *A* who is capable of breaking the IND-sMIBSC-CCA security with a non-negligible advantage 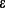. *B* makes use of *A* to solve the DBDH instance. *B* simulates the system with various oracles *H*_1_, *H*_2_, *H*_3_ and *H*_4_ and allows *A* to make polynomially bounded number of queries, adaptive to these oracles. The game between *A* and *B* is demonstrated below:

#### Setup

*B* sets *P*_1_ = *cP*, *P_pub_ = bP*, and gives <*G*_1_,*G*_2_,*q*,*e*,*P*,*P_pub_*,*H*_1_,*H*_2_,*H*_3_,*H*_4_> to the attacker *A* as the public parameters. Upon receiving the system parameters, *A* outputs *n* target identities 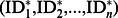.

#### Phase 1

*A* performs a number of queries to *B*:

Let *H*_1_, *H*_2_, *H*_3_ and *H*_4_ be random oracles controlled by *B* as follows. The results of querying *H*_1_, *H*_2_, *H*_3_ and *H*_4_ are stored in *H*_1_-list, *H*_2_-list, *H*_3_-list and *H*_4_-list, respectively.

#### *H*_1_-query

Input an identity 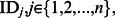 to *H*_1_. If there exists (ID*_j_*, *l_j_*, *Q_j_*) in *H*_1_-list, *B* returns *Q_j_*; othzerwise, does as follows:

1. Choose an integer 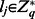 at random;
2. If 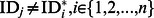, compute *Q_j_* = *l_j_P*; otherwise, compute *Q_j_* = *l_j_P*-*P*_1_;
3. Put (ID*_j_*, *l_j_*, *Q_j_*) into *H*_1_-list;
4. Return *Q_j_*.

#### *H_i_*-query


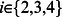
: To answer these inquiries, *B* searches the corresponding list *H_i_*-*list*(*i* = 2,3,4). If the corresponding answer has existed already, *B* returns the answer to *A*. Otherwise, *B* randomly chooses an element in proper scope as the result and returns it to *A*, and at the same time, *B* adds the inquiry and the result into the corresponding list.

#### Extraction query

Upon receiving private key extraction query on identity ID*_j_* 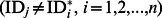, *B* searches for (ID*_j_*, *l_j_*, *Q_j_*) in *H*_1_-list. *B* recovers triple (ID*_j_*, *l_j_*, *Q_j_*) in *H*_1_-query and computes his private key *D* = *l_j_P_pub_* = *l_j_bP*, and returns it to *A*. If 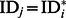, *B* aborts and outputs “failure”.

#### Anony-signcryption query

Upon receiving *A*’s anony-signcryption query (*M*, ID*_S_*, *L*), *B* checks if 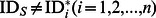. If 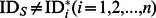, *B* shall get the private key of ID*_S_* through the Extraction query. After that, *B* can run the Anony-signcryption query to generate the ciphertext *M*. An alternative to this is:

1. *B* randomly chooses two secret integers 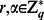, and then computes *Y* = *rl_s_P*, 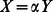, 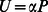, 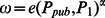 and 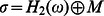.
2. For *i* = 1, 2, …,*n*, compute *x_i_* = *H*_3_(ID*_i_*) and 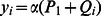, where *Q_i_* is the public key of ID*_i._*
3. For *i* = 1,2,…,*n*, compute: 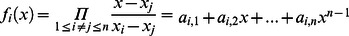, where 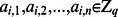.
4. For *i* = 1,2,…,*n*, compute 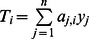 and then let *T* = {*T*_1_,*T*_2_,…,*T_n_*}.
5. *B* randomly chooses an integer 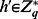, where 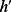 is set as the output for the random oracle query 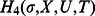
(This is possible because the random oracles are manipulated by *B*). Then, *B* computes 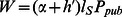.
6. *B* gets the ciphertext 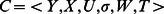 and returns it to *A*.

#### De-signcryption**** query

On receiving the De-signcryption query of the ciphertext *C* together with an identity ID*_j_*, *B* proceeds as follows:

1. If 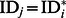, *B* shall return that the ciphertext *C* is invalid because *B* does not know the private key of ID.
2. If 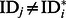, *B* computes 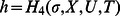, and verifies whether the equation *e*(*W*, *P*) = *e*(*X*+*hY*, *P_pub_*) holds. If it does not hold, the ciphertext is not valid and then *B* outputs 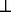. If it holds, *B* does the following steps:
  - Find the secret key *D* corresponding to ID from the *H*_1_-list.
  - Compute 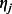 according to *T*.
  - Compute 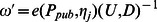 and then get the plaintext as 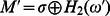.

If all the above verifications are true, then *B* outputs the message 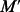. Otherwise, the ciphertext is invalid, and *B* outputs 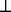.

#### Challenge

*A* outputs a target plaintext pair (*M*_0_, *M*_1_) and a private key *D_S_*. Upon receiving (*M*_0_, *M*_1_) and *D_S_*, *B* picks up a random bit 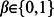 and signcrypts the message 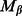. Firstly, *B* searches *H*_1_-list to get 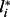 related to 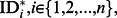 and their public key 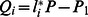, then computes 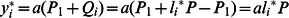 to get 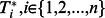. *B* finally creates the target ciphertext 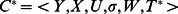 where 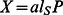, 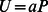, 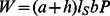 and 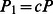, and then returns *C* ^*^ to *A*.

#### Phase 2

*A* performs a number of queries as Phase 1. Note that *A* cannot query the identity information of 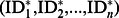 in extraction query, and cannot query *C* ^*^ in de-signcryption query.

#### Guess

Finally, *A* outputs its guess 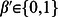. If 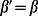, *B* wins this game and outputs 1 as the answer of DBDH problem because 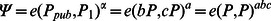. Otherwise, *B* outputs 0.

From the above discussion, we shall analyze the advantages of *B* in the following. For *q_d_* de-signcryption queries, the probability to reject a valid ciphertext is not greater than 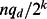. If *A* wins the IND-sMIBSC-CCA game, the advantage of *B* is
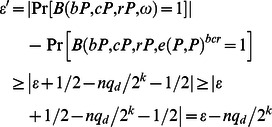



#### Theorem 5

*In the* SUF-sMIBSC-CMA *security model,* if there is an adversary F who can win the game in the time t with a non-negligible advantage 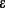 as described in the definition 5, there will exist an algorithm B which can solve the CDH problem in the time 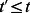 with an advantage 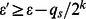, where F can ask at most q_e_ extraction queries, q_s_ anony-signcryption queries and 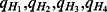 queries to H_1_, H_2_, H_3_, H_4_, respectively.

#### Proof

The challenge *B* is given (*P*,*aP*,*bP*) as an instance of the CDH problem. Assume that there is an adversary *F* who has a non-negligible advantage 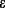 in breaking the SUF-sMIBSC-CMA security. Then, *B* uses *F* to solve the CDH problem. Firstly, *B* simulates the system with the various oracles *H*_1_, *H*_2_, *H*_3_ and *H*_4_, and then allows *F* to adaptively ask polynomially bounded number of queries to these oracles. The game between *B* and *F* is demonstrated below:

#### Setup

*B* sets *P_pub_ = bP*, and gives <*G*_1_, *G*_2_, *q*, *e*, *P*, *P_pub_*, *H*_1_, *H*_2_, *H*_3_, *H*_4_> to the attacker *F* as the public parameters. Upon receiving the system parameters, *F* outputs *n* target identities 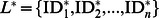.

#### Attack

*F* adaptively performs polynomially bounded number of queries to the various oracles in this phase, which are similar to those in Theorem 4.

#### Forgery

*F* generates a target ciphertext 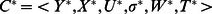. If the forgery is successful, the equation 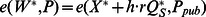 holds. Define Q_S_^*^ = l_S_^*^P = *aP*, and then we have 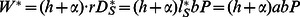. Now, it will be very easy to extract the CDH problem’s solution 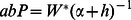.

We consider the advantage of *F*’s success here. As in the anony-signcryption query, the probability for *B* to answer a failure signcryption query is not greater than *q_s_*/2*^k^*, and then the advantage is 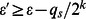.

#### Theorem 6

*In the* ANON-sID-CCA *security model, if an adversary A has advantage* 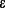 *against the game defined in Definition 6 within running time t (where* A mak*es* at most q_e_ private key extraction queries, q_s_ *anony-signcryption queries,* q_d_ de*-sign*cryption queries and 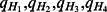 queries to the Hash functions H_1_,H_2_,H_3_ and H_4_, *respectively), then there is an algorithm* B *in solving the DBDH-M problem with an advantage 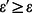.*

#### Proof

The challenger *B* is challenged with an instance 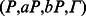 of the DBDH-M problem. Assume that there is an adversary *A* who is capable of breaking the ANON-sID-CCA security with a non-negligible advantage 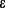. *B* makes use of *A* to solve the DBDH-M instance. *B* simulates the system with hash functions *H*_1_, *H*_2_, *H*_3_ and *H*_4_, and allows *A* to make polynomially bounded number of queries. The game between *B* and *A* is demonstrated below:

#### Phase 1

Suppose that *A* outputs a target identity pair (ID_1_^*^, ID_2_^*^).

#### Setup

*B* sets the public key 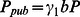 and lets 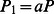, where 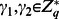, and *aP* and *bP* are given from the instance of the DBDH-M problem. Here, *B* does not know *a* and *b*. *A* performs polynomially bounded number of queries to *H*_1_, *H*_2_, *H*_3_ and *H*_4_, which are similar to those in Theorem 4.

#### Phase 2

Upon receiving the private key extraction query of an identity ID*_j_* such that 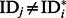, for 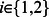, according to *Q_j_* = *l_j_P* = *aP*, *B* computes *D_j_* = *l_j_P_pub_*.

#### Phase 3

Upon receiving a decryption query about (*C* ^*^, ID*_i_* ^*^) *i* = 1,2 and 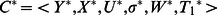. Challenger *B* performs the following steps:

1. Compute 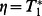.
2. Compute 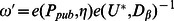.
3. Judge whether 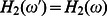 holds. If not, return “reject” to *A*, otherwise return 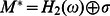 to *A*.

#### Challenge

*A* outputs a target plaintext *M*. Upon receiving *M*, *B* does the following steps:

1. Compute 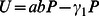.
2. Compute 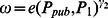.
3. Create a target ciphertext 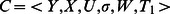 and return it to *A*.

#### Phase4

*A* issues private key extraction queries as those in Phase 2 and decryption queries for target identities as those in Phase 3, where a restriction here is that 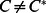.

#### Guess

Finally, *A* outputs its guess 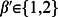. If 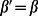 holds, *B* outputs 1. Otherwise, *B* outputs 0. If *T*_1_ = *a* ^2^ *bP*, then
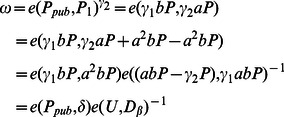
(7)


Thus, we have 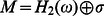.

Here, *C* is the valid ciphertext. We can get 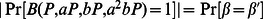 where 
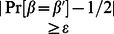
, and 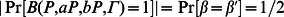 where 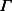 is randomly chosen from *G*_1_. Therefore, we have 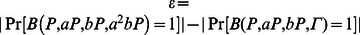 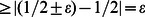, that is, 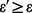.

### Efficiency Analysis

We compare our scheme with existing signcryption schemes [9], [10], [11], [12], [13], [15], [17], [19] in terms of calculation costs and communication traffic (ciphertext length). In order to facilitate the description, we define the following symbols shown in Table 2:

**Table 2:** Symbol Definition.

Symbols	Meaning
*T_p_*	Time for bilinear pair operation
*T_a_*	Time for addition operation
*T_m_*	Time for scalar multiplication operation
*T_e_*	Time for exponentiation operation
*T_h_*	Time for Hash operation
*T_s_*	Time for symmetric encryption algorithm

First, we talk about the signcryption process. In the proposed scheme, the operation about Lagrange interpolation can also be pre-processed, so these operations can be excluded when considering computational complexity. In order to signcrypt a message *M*, our scheme needs 1 bilinear operation, 2 addition operations in *G*_1_, 6 scalar multiplications in *G*_1_, 1 exponentiation in *G*_2_ and 2 hash operations. The length of the ciphertext is (*n*+4)|*G*_1_|+|*M*|. The specific comparison results are shown in Table 3, from which one can see that our scheme performs much better than most of the existing schemes in terms of number of parameters, computation complexity and the ciphertext length.

**Table 3:** Signcryption Efficiency Comparison.

Schemes	Number of parameters	Computation Complexity	Ciphertext length
Yu *et al*.’s [9]	10	*T_p_*+(*n*+1)*T_a_*+(*n*+5)*T_m_*+*T_e_*+2*T_h_*	(*n*+2)|*G*_1_|+|*G*_2_|+|*M*|+*n*|ID|
Shamila *et al*.’s [10]	*n*+9	(*n*+1)*T_a_*+(*n*+3)*T_m_*+*T_e_*+2*T_h_*	3|*G*_1_|+|*M*|+*n*|ID|
Elkamchouchi [11]	8	2*T_p_*+(*n*+1)*T_a_*+(*n*+4)*T_m_*+2*T_e_*+2*T_h_*+ *T_s_*	(*n*+2)|*G*_1_|+|*M*|+*n*|ID|+|*Z_q_*|
Pang *et al*.’s [12]	12	*T_p_*+(3*m*−2)*T_a_*+(2*m*+1)*T_m_*+*T_e_*+(*m*+1)*T_h_*	(*m*+*n*+2)|*G*_1_|+|*M*|+*m*|ID|
Lal *et al*.’s [13]	11	(3*m*+*n*−2)*T_a_*+(2*m*+*n*+2)*T_m_*+*T_e_*+(*m*+2)*T_h_*	(*m*+*n*+2)|*G*_1_|+|*M*|+(*m+n*)|ID|
Zhang B *et al*.’s [15]	13	*T_p_* +(*m*+*n*+1)*T_m_*+(2*m*+*n*+3)*T_e_* +2*T_h_*	(*m*+*n*+2)|*G*_1_|+|*M*|+*m*|ID|
Zhang J *et al*.’s [17]	10	*T_p_* +(4*m*)*T_m_*+(4*m*−2)*T_a_*+(*m*+2)*T_h_*	(*m*+2)|*G*_1_|+|*M*|
Huang *et al*.’s [19]	10	*T_p_*+ (2*m*−3)*T_a_*+(2*m*+2)*T_m_* +(*m*+2)*T_h_*	2|*G*_1_|+*m*|*G*_2_|+2|*M*|+*m*|ID|+*m*|*Z_q_*|
Ours	10	*T_p_*+2*T_a_*+6*T_m_*+*T_e_*+2*T_h_*	(*n*+4)|*G*_1_|+|*M*|

|*G*_1_|: the length of the elements in *G*_1_; |ID|: the length of ID; |*M*|: the length of the plaintext *M*;

*m*: the number of signers (*m* = 1 in schemes [9]–[11] and our scheme); *n*: the number of recipients.

Regarding the de-signcryption in our scheme, some calculations of the de-signcryption algorithm are used to judge the validity of ciphertext and the authorization of the recipient, which is important for broadcast-based communications to avoid receiving unwanted information (e.g. SPAM). Note that although the schemes [9], [10], [11], [13], [15] directly provide the recipients’ true identities in the ciphertext, in fact the recipient cannot absolutely ensure whether he/she is authorized before checking the validity of the ciphertext. The number of pair operations (*T_p_*, the most time-consuming operation in the existing schemes and our scheme) in our decryption algorithm is smaller than those of the existing schemes, which makes our scheme more attractive in terms of computation performance. Table 4 shows a comparison between the proposed scheme and the existing ones [9], [10], [11], [12], [13], [15], [17], [19].

**Table 4:** De-signcryption Efficiency Comparison.

Schemes	Ciphertext validity or integrity	Authorized or not	Decryption
Yu *et al*.’s [9]	3*T_p_*+2*T_a_*+3*T_m_*+3*T_h_*	3*T_p_*+2*T_a_*+3*T_m_*+3*T_h_*	3*T_p_*+2*T_a_*+3*T_m_*+3*T_h_*
Shamila *et al*.’s [10]	3*T_p_*+2*T_a_*+(3*n* +3)*T_m_*+2*T_e_*+(*n*+1)*T_h_*	3*T_p_*+2*T_a_*+(3*n* +3)*T_m_*+2*T_e_*+(*n*+1)*T_h_*	3*T_p_*+2*T_a_*+(3*n* +3)*T_m_*+2*T_e_*+(*n*+1)*T_h_*
Elkamchouchi *et al*.’s [11]	2*T_p_*+*T_e_*+*T_m_*+2*T_h_*	2*T_p_*+*T_e_*+*T_m_*+2*T_h_*	4*T_p_*+2*T_a_*+*T_e_*+3*T_h_* +*T_s_*
Pang *et al*.’s [12]	2*T_p_*+ (2*m*−1)*T_a_*+*mT_m_*+ *mT_h_*	(2*m*−1)*T_a_*+(*m*+2)*T_m_*+*mT_h_*	4*T_p_*+(2*m*+*n*−2)*T_a_*+(*m*+*n*+1)*T_m_*+(*m*+2)*T_h_*
Lal *et al*.’s [13]	2*T_p_*+ (2*m*−1)*T_a_*+*mT_m_*+*mT_h_*	4*T_p_*+2*mT_a_*+(*m*+1)*T_m_*+(*m*+1)*T_h_*	4*T_p_*+2*mT_a_*+(*m*+1)*T_m_*+(*m*+1)*T_h_*
Zhang B *et al*.’s [15]	(*m+*5)*T_p_*++*T_a_*+(*m*+*|M|*+2)*T_m_*+2*T_h_*	(*m+*5)*T_p_*++*T_a_*+(*m*+*|M|*+2)*T_m_*+2*T_h_*	(*m+*5)*T_p_*++*T_a_*+(*m*+*|M|*+2)*T_m_*+2*T_h_*
Zhang J *et al*.’s [17]	4*T_p_*+2*mT_a_*+*mT_m_*+(*m*+2)*T_h_*	N/A	4*T_p_*+2*mT_a_*+*mT_m_*+(*m*+2)*T_h_*
Huang *et al*.’s [19]	3*T_p_* +(*m*+1)*T_a_*+2*mT_m_*+(*m*+2)*T_h_*	N/A	3*T_p_* +(*m*+1)*T_a_*+2*mT_m_*+(*m*+2)*T_h_*
Ours	2*T_p_*+ *T_a_*+*T_m_*+*T_h_*	2*T_p_*+*T_a_*+*T_m_*+*T_h_*	2*T_p_*+ *nT_a_*+(*n*−1)*T_m_*+2*T_h_*

|*M*|: the length of the plaintext *M*; *m*: the number of signers (*m* = 1 in schemes [9]–[11] and our scheme); *n*: the number of recipients.

Note: N/A refers to a single-recipient scheme where the message is transmitted using a unicast communication channel, thus it is unnecessary for the recipient to judge whether he/she is authorized.

### Discussion of Merit and Demerit

Compared to existing schemes, our scheme has some advantages. To achieve the signer’s anonymity, the identity of the signer is no longer included in the ciphertext, although the private key of the signer is necessary for signcryption. The recipients can therefore only judge if the ciphertext received is from a trusted signer, but they cannot determine the real identity of the signer. To achieve the recipient’s anonymity, the ID information of all authorized recipients is mixed by the Lagrange interpolation polynomial during the signcryption process, which prevents the recipient’s ID from being exposed. This method also ensures that only the recipient, who has got the entire ciphertext, can decrypt the ciphertext, thus achieving the decryption fairness. The ID-based cryptography enables one user to confidentially send messages to other users, despite of whether the latter is a registered user, and the public verification property of our scheme enables unregistered users to judge the validity of the received ciphertext before having to register himself/herself with PKG. The merit/demerit comparison between the existing schemes and our scheme is summarized in Table 5.

**Table 5:** Comparison of merits and demerits.

Scheme	Signer anonymity	Recipient anonymity	Decryption fairness	Public Verification
Yu *et al*.’s [9]	No	No	No	No
Shamila *et al*.’s [10]	No	No	No	No
Elkamchouchi *et al*.’s [11]	No	No	No	No
Pang *et al*.’s [12]	Yes (*)	Yes	Yes	No
Lal *et al*.’s [13]	Yes (*)	No	No	No
Zhang B *et al*.’s [15]	Yes (*)	No	No	No
Zhang J *et al*.’s [17]	Yes (*)	No	No	No
Huang *et al*.’s [19]	Yes (*)	No	No	No
Ours	Yes	Yes	Yes	Yes

**Note:** (*) refers to schemes prone to the *cross-comparison attack* and *joint conspiracy attack*.

From Table 5, we can see: (1) The schemes [12], [13], [15], [17], [19] have taken anonymity of the sender into account. However, they are all prone to the *cross-comparison attack* and *joint conspiracy attack*. In these schemes, in order to protect the privacy of the sender, the sender randomly chooses some legitimate participants to hide the true identity. But in practice, these schemes are vulnerable to the *cross-comparison attack* and *joint conspiracy attack* mentioned above. (2) The schemes [9], [10], [11] cannot assure the anonymity of the sender because the identity of the sender is directly given in the ciphertext. (3) The schemes [9], [10], [11], [13], [15], [17], [19] cannot assure the anonymity of the recipient. In these schemes, the ciphertext includes two parts: a recipient identity list and each recipient’s specific data. A recipient identity list is required so that an authorized recipient is able to find his/her specific data required for decryption of the ciphertext. Because the recipient identity list is given in plaintext, the ID information of each recipient is exposed, and thus the anonymity of recipients is not assured. This has the advantage that, as long as an authorized recipient receives his/her specific data correctly, he/she can decrypt the ciphertext to retrieve the corresponding message even if other recipients’ specific data are invalidated during transmission. While this seems to represent an advantage on the first sight, it also represents a problem regarding decryption fairness. Decryption unfairness can cause the sender to cheat some recipients actively by just sending incorrect recipient-specific data. (4) In all the existing schemes, public verification is not considered because the identity of the sender or the recipient must be given in the ciphertext in plaintext form, thus there are no requirements for public verification. But in a completely anonymous scheme, public verification is a necessity for recipients so that receiving or operating on unwanted messages is prevented.

To summarize, the ciphertext in our scheme no longer contains the real identity information of all participants, thus our scheme meets anonymity of the sender and recipients at the same time, and efficiently protects the privacy of all involved participants. Even more important, this scheme possesses fair decryption and public verification properties. Furthermore, our scheme is easy to implement in exsiting applications. Here, we also take a network conference application as an example. In such a case, a message sender needs only to transform the plaintext message to a ciphertext message using our encryption algorithm and then broadcasts it through the broadcast communication channel, while a message recipient simply needs to decrypt the ciphertext using our decryption algorithm. Our scheme requires only extra encryption or decryption operations for each participant and leaves the original implementation untouched, which in fact should represent an easy implementation of our scheme. While our scheme has the advantages mentioned above, it also has some disadvantages, namely its application, which increases the costs for the implementation. For example, it probably takes a great deal to establish PKG and maintain it, which may affect routine application of our scheme to some extent.

### Conclusions

Due to the nature of broadcasting communications, the anonymity of both the sender and the recipient is of upmost importance in multi-recipient signcryption. However, almost none of the existing multi-recipient signcryption schemes take the anonymity of recipients into account. Although there are several schemes that provide a solution to the anonymity of the signer, they have known limitations. Owing to practical application requirements, a completely anonymous multi-recipient signcryption becomes more and more important. Aiming at the participants’ anonymity, a completely anonymous multi-recipient signcryption is proposed in this paper. The new scheme ensures anonymity of all participants, the sender and all recipients. Furthermore, each recipient can easily judge whether the received message is from an authorized source, but he/she cannot determine the true identity of the sender. Each recipient can easily judge whether he/she is an authorized recipient, but he/she cannot determine the identity of any other authorized recipient. At the same time, the validity of the ciphertext can be verified publicly. The confidentiality, unforgeablity and anonymity of our scheme are formally proven using the random oracles model. Compared to existing schemes, our scheme is more efficient in computation and ciphertext length, and possesses more merits, which makes our scheme suitable for practical applications. Our scheme is important in group-oriented network applications, such as the network conference, paid-TV or DVD broadcasting. The proposed scheme solves the secure communication problem among authorized participants, while at the same time, it provides complete anonymity for all involved participants.
